# CagL polymorphisms D58/K59 are predominant in *Helicobacter pylori* strains isolated from Mexican patients with chronic gastritis

**DOI:** 10.1186/s13099-019-0286-9

**Published:** 2019-02-13

**Authors:** Adolfo Román-Román, Verónica I. Martínez-Santos, Carlos A. Castañón-Sánchez, Alan J. Albañil-Muñoz, Paola González-Mendoza, Diana G. Soto-Flores, Dinorah N. Martínez-Carrillo, Gloria Fernández-Tilapa

**Affiliations:** 1 Laboratorio de Investigación en Bacteriología, Facultad de Ciencias Químico-Biológicas, Universidad Autónoma de Guerrero, Av. Lázaro Cárdenas s/n C.U. Sur., C.P. 39090 Chilpancingo, Guerrero Mexico; 2 Universidad Autónoma de Guerrero, Av. Javier Méndez Aponte No. 1, Fracc. 10, Col. Servidor Agrario, C.P. 39070 Chilpancingo, Guerrero Mexico; 3 Hospital Regional de Alta Especialidad de Oaxaca, Aldama s/n, Col. Centro, C.P. 71256 San Bartolo Coyotepec, Oaxaca Mexico; 4 Laboratorio de Investigación Clínica, Facultad de Ciencias Químico-Biológicas, Universidad Autónoma de Guerrero, Av. Lázaro Cárdenas s/n C.U. Sur., C.P. 39090 Chilpancingo, Guerrero Mexico

**Keywords:** *H. pylori*, *cagL* polymorphisms, Polymorphic region, Chronic gastritis

## Abstract

**Background:**

*Helicobacter pylori* is a Gram-negative bacterium that colonizes the gastric mucosa in humans. One of the main virulence factors of *H. pylori* is the *cag* pathogenicity island (*cag*PAI), which encodes a type 4-secretion system (T4SS) and the cytotoxin CagA. Translocation of CagA through the T4SS triggers host-signaling pathways. One of the T4SS proteins is CagL, which is necessary for CagA translocation. CagL is a 26-kDa protein that contains a hypervariable motif, which spans residues 58 to 62. Several polymorphisms in this region have been associated with different disease outcomes, e.g. in Mexico, N58 is associated with a higher risk of gastric cancer. The aim of this work is to analyze the sequence of the hypervariable motif (residues 58 to 62) of clinical isolates from Mexican patients with chronic gastritis, and to correlate these polymorphisms with the *vacA* genotype.

**Results:**

Of the 164 biopsies analyzed, only 30.5% (50/164) were positive for *H. pylori*. Thirty-six of the 50 clinical isolates (72%) were *cagA* positive, and 40 (80%) had the most virulent *vacA* genotype (s1/m1). Of the *cagA* positive strains, 94.4% were *vacA* s1/m1. All the *cagA*^+^ strains contained the *cagL* gene. The most prevalent sequence in the polymorphic region (residues 58–62) was DKMGE (75.8%, 25/33), followed by NKMGQ and NEIGQ (6.1%, 2/33), and DEIGQ, NKMGE, DKIGE, and DKIGK (3%, 1/33). Regarding polymorphisms in positions 58 and 59, the most common were D58/K59 (81.8%, 27/33), followed by N58/K59 (9.1%, 3/33), and D58/E59 (3%, 1/33). Only two isolates (6.1%) contained residues N58/E59, which correspond to those found in *H. pylori* strain ATCC 26695. 92.6% of the clinical isolates having polymorphism D58/K59 had the genotype *vacA* s1/m1, considered to be the most virulent, while 7.4% had the genotypes *vacA* s1/m2 and s2/m2.

**Conclusions:**

In Mexican patients, CagL polymorphisms D58, K59, M60, E62, K122, and I134 are more common in patients with chronic gastritis.

## Background

*Helicobacter pylori* is a Gram-negative, spiral-shaped bacterium that colonizes the gastric mucosa in humans. In many cases, it can persist without causing symptoms, although in some cases it can produce chronic gastritis. The persistent colonization of the gastric mucosa by *H. pylori* can lead to the development of gastroduodenal diseases like peptic ulcer, gastric lymphoma, and gastric cancer [1]. *H. pylori* has two main virulence factors, the *cag* pathogenicity island (*cag*PAI) and the pore forming toxin *vacA*, whose presence or absence identifies highly virulent (type-1) from less virulent (type-2) strains, respectively, thus being strong predictors of severe disease outcome [2, 3]. The *cag*PAI is a 40-kb chromosomal region that contains 27–31 genes that encode the type IV secretion system (T4SS) and an effector protein [4]. The T4SS forms a needle-like surface appendage called the T4SS pilus, which is induced upon contact with the host cell membrane [5]. The effector protein CagA is encoded by the *cagA* gene. This protein is translocated into the cytoplasm of the gastric epithelial cells through the T4SS, where it is phosphorylated at the tyrosine residues of the EPIYA motifs, causing multiple cellular alterations [6, 7]. One of the genes that encode the T4SS is *cagL*, which encodes for the 26-kDa protein CagL. It has been shown that this protein is localized on the surface of the T4SS of *H. pylori* and in intracellular pools, is necessary for CagA translocation, and interacts with CagI [5, 8]. It also contributes to transient hypochlorhydria by disrupting the interaction between the integrin and metalloprotease ADAM17 and the integrin α_5_β_1_, thus activating NF-κB-mediated repression of the gastric H, K-adenosine triphosphatase α-subunit (HKα) [9].

The role of some regions or motifs of CagL in different phenotypes has been assessed. For example, its C-terminal coiled-coil region is involved in IL-8 secretion, host cell elongation, and binding of the T4SS to the host cell [10]. CagL also contains an arginine–glycine–aspartate (RGD) motif at residues 76–78, located within the second large α-helix [11], which is essential for binding to the human integrin α5β1 receptor [5], as well as integrins α_v_β_5_ and α_V_β_3_ [12, 13], although it can also bind to human fibronectin in a RGD independent manner [14]. This motif is also responsible of CagL fibronectin-like effect on host cells, since it is involved in cell spreading triggering, formation of focal adhesions, and activation of several tyrosine kinases [15]. The RGD motif is right next to the sequence LXXL, of which, the two-leucine residues (L79 and L82) contribute to the adhesion to different cell lines through interaction with integrin α_V_β_6_ [16]. CagL also contains a second motif named RHS (RGD helper sequence), which contains residues phenylalanine–glutamic acid–alanine–asparagine–glutamic acid (FEANE). This motif also contributes to CagL binding to integrins [17]. The third important region in CagL is the CagL hypervariable motif (CagLHM), which spans residues 58 to 62 [18]. It is located in an unresolved flexible region between helices α1 and α2 [19]. Although its function is still controversial, it has been shown that certain polymorphisms correlate with diseases in a geographical-dependent manner. For example, polymorphism Y58/E59 is associated with gastric cancer in patients from Taiwan, while in India the polymorphism associated with this disease is D58/K59 [20, 21]. In Mexico, only the polymorphism N58 has been associated with a higher risk of gastric cancer [22]. So far, 33 combinations of polymorphisms in the hypervariable motif have been identified worldwide, the most common being DKMGE, NEIGQ, NKIGQ, and DKIGK. Of these, all are found in North and South American strains, except for DKIGK, which is more prevalent in East/Southeast Asia/Australasia [23]. The aim of this work was to analyze the sequence of the hypervariable motif (residues 58 to 62) of clinical isolates from Mexican patients with chronic gastritis, and to correlate these polymorphisms with the *vacA* genotype of *H. pylori*.

## Results

### *Helicobacter pylori* prevalence

**Fig. 1:**
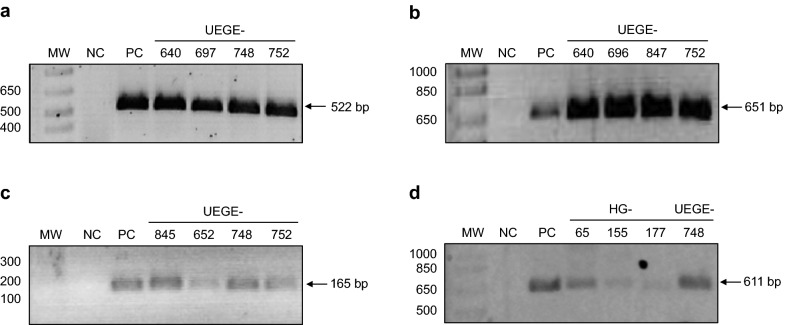
Representative gels of PCR amplified fragments. **a** 16S RNA fragment. Lanes: MW—molecular weight marker (bp), NC—negative control, PC—positive control (DNA from strain ATCC 43504), 4 to 7—clinical isolates UEGE-640, UEGE-697, UEGE-748, and UEGE-752. **b** *cagL* fragment amplified with the first set of primers (651 bp). Lanes: MW—molecular weight marker (bp), NC—negative control, PC—positive control (DNA from strain ATCC 43504), 4 to 7—clinical isolates UEGE-640, UEGE-696, UEGE-847, and UEGE-752. **c** *cagL* fragment amplified with the second set of primers (165 bp). Lanes: MW—molecular weight marker (bp), NC—negative control, PC—positive control (DNA from strain HP26695), 4 to 7—clinical isolates: UEGE-845, UEGE-652, UEGE-748, and UEGE-752. **d** *cagL* fragment amplified with the third set of primers (611 bp). Lanes: MW—molecular weight marker (bp), NC—negative control, PC—positive control (DNA from strain HP26695), 4 to 7—clinical isolates: HG-65, HG-155, HG-177, and UEGE-748

We analyzed 164 patients with histopathological diagnosis of chronic gastritis, of which 61.6% (101/164) were female, and 38.4% (63/164) were male. The mean age of the patients was 48 years old (± 17), ranging between 19 and 89 years old. The frequency of *H. pylori* isolation was 30.5% (50/164). All strains identified as *H. pylori* by culture were confirmed by PCR amplification of a fragment of the 16S rRNA gene (Fig. 1a).

Thirty-six of the 50 clinical isolates (72%) were *cagA* positive, and 40 (80%) had the most virulent *vacA* genotype (s1/m1). Of the *cagA* positive strains, 94.4% were *vacA* s1/m1 (Table 1). In the *cagA* negative isolates, absence of the *cag*PAI was confirmed by empty site PCR.

**Table 1:** Frequency of *cagA* and *vacA* genotypes in clinical isolates of *H. pylori* from patients with chronic gastritis

	*cagA*	
	Positive	Negative
	n = 36 (100%)	n = 14 (100%)
*vac*A		
s1/m1	34 (94.4%)	6 (42.9%)
s1/m2	1 (2.8%)	0
s2/m2	1 (2.8%)	8 (57.1%)

### Prevalence of *cagL*

A PCR product of 651 bp was amplified in 24 of the 36 *cagA* positive clinical isolates (Fig. 1b) using primers cagL sense and cagL antisense (Table 4). To determine if the 12 remaining strains were *cagL* negative, we performed a second PCR using primers cagL-Fwd-2 and cagL-16 (Table 4). A product of 165 bp was amplified in the 12 strains (Fig. 1c), indicating that all *cagA* positive strains contain the *cagL* gene. In order to obtain sequences suitable for uploading in the GenBank (> 200 bp), the DNA from those strains that were positive with the second set of primers was subjected to a third PCR using primers cagL-Fwd-2 and cagL antisense. With the new combination of primers, a product of 611 bp was amplified in 9 of the 12 strains (Fig. 1d), and the 33 PCR products amplified were sequenced.

### CagL polymorphisms

**Fig. 2:**
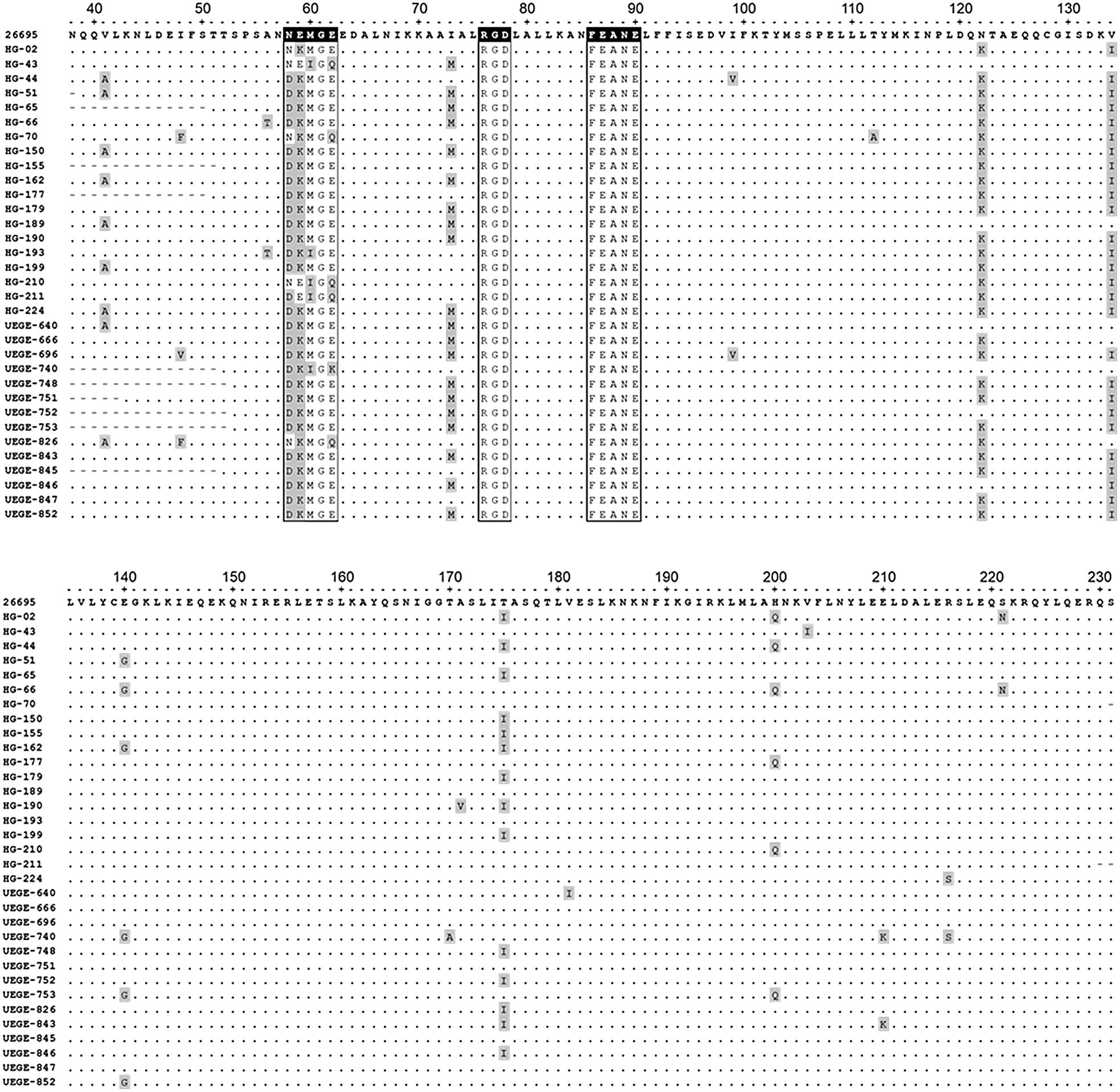
Sequence alignment of CagL from 33 clinical isolates registered in GenBank. Sequence from strain ATCC 26695 was used as reference. CagLHM, RGD, and RHS (FEANE) motifs are indicated in squares, black squares indicate the polymorphic region (residues 58 to 62), and the RDG and RHS motifs in the reference sequence. Polymorphisms are shown in gray. Accession numbers: MG051618-MG051641 and MG214979-MG214987

The sequences of 33 of the 36 *cagL*^+^ clinical isolates were aligned with the sequence of strain ATCC 26695, showing a high level of conservation (Fig. 2). Motifs RGD and RHS are 100% identical in all strains.

**Fig. 3:**
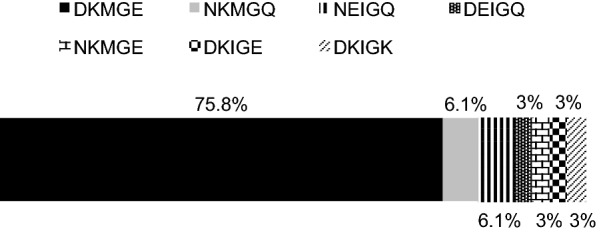
Frequency of sequences in the CagLHM motif (positions 58 and 62). Sequence DKMGE was found in 25 of 33 clinical isolates, sequences NKMGQ and NEIGQ in 2, and sequences DEIGQ, NKMGE, DKIGE, and DKIGK in 1 clinical isolate each. Frequencies are shown in percentages

The most prevalent sequence in the polymorphic region (residues 58–62) was DKMGE (75.8%, 25/33), followed by NKMGQ and NEIGQ (6.1%, 2/33, respectively), DEIGQ, NKMGE, DKIGE, and DKIGK (3%, 1/33, respectively) (Fig. 3). One clinical isolate had four polymorphisms (3%) (UEGE-740), two had three polymorphisms (6.1%) (HG-193 and HG-211), one had only one polymorphism (3%) (HG-02), and the rest (87.9%) had two (Fig. 2). Interestingly, one of the clinical isolates with three polymorphisms (HG-193), and the one with four (UEGE-740) had the sequence DKI, which is more prevalent in East-Asian strains [24].

**Fig. 4:**
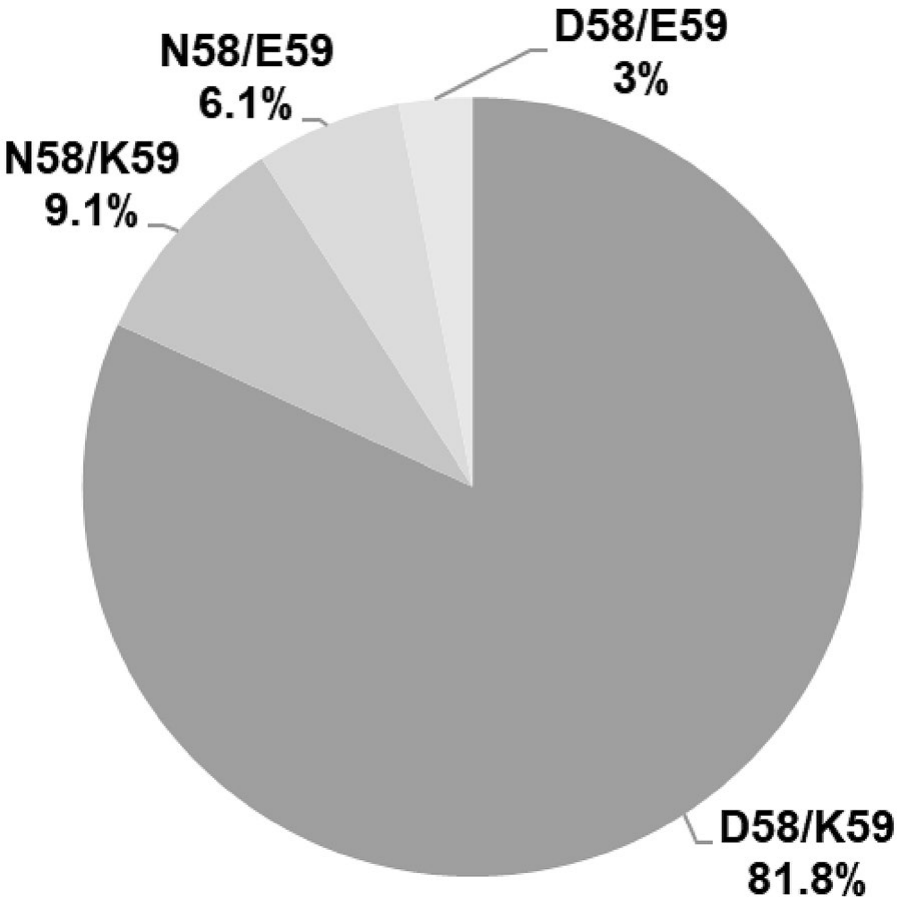
Frequency of CagL polymorphisms in positions 58 and 59. Polymorphism D/K was found in 27 clinical isolates, polymorphism N/K in 3, polymorphism N/E in 2, and polymorphism D/E in 1. Frequencies are shown in percentages

Regarding polymorphisms in positions 58 and 59, the most common were D58/K59 (81.8%, 27/33), followed by N58/K59 (9.1%, 3/33), and D58/E59 (3%, 1/33) (Fig. 4). Only two isolates (6.1%) contained residues N58/E59 (HG-43 and HG-210), which correspond to those found in *H. pylori* strain ATCC 26695.

Our sequence alignment (Fig. 2) revealed that, besides the polymorphisms found in the CagLHM motif, there were other 19 polymorphisms both upstream and downstream of this region (Table 2). The most common were A41, M73, K122, I134, and I175, being found in > 9 strains; while the rest (F48, V48, T56, V99, A112, G140, A170, V171, I181, Q200, I203, K210, S216, and N221) were identified in < 6 strains.

**Table 2:** CagL polymorphisms in strains isolated from patients with chronic gastritis

Residue in 26695	Polymorphism	Frequency n (%)	Strain (s)	CagL region
V41	A	9/33 (27.3%)	HG-44, -51, -150, -162, -189, -199, -224 UEGE-640, -826	α1
I48	F	2/33 (6%)	HG-70, UEGE-826	L1
	V	1/33 (3%)	UEGE-696	
A56	T	2/33 (6%)	HG-66, HG-193	L1
N58	D	28/33 (84.8%)	HG-44, -51, -65, -66, -150, -155, -162, -177, -179, -189, -190, -193, -199, -211, -224, UEGE-640, -666, -696, -740, -748, -751, -752, -753, -843, -845, -846, -847, -852	L1
E59	K	30/33 (90.9%)	HG-02, -44, -51, -65, -66, -70, -150, -155, -162, -177, -179, -189, -190, -193, -199, -224 UEGE-640, -666, -696, -740, -748, -751, -752, -753, -826, -843, -845, -846, -847, -852	L1
M60	I	5/33 (15.1%)	HG-43, -193, -210, -211, UEGE-740	L1
E62	Q	5/33 (15.1%)	HG-43, -70, -210, -211 UEGE-826	α2
	K	1/33 (3%)	UEGE-740	
I73	M	20/33 (60.6%)	HG-43, -51, -65, -66, -150, -162, -179, -189, -190, -224, UEGE-640, -666, -696, -748, -751, -752, -753, -843, -846, -852.	α2
I99	V	2/33 (6%)	HG-44, UEGE-696	α2
T112	A	1/33 (3%)	HG-70	α3
N122	K	27/33 (81.8%)	HG-02, -44, -51, -65, -66, -70, -150, -155, -162, -177, -179, -190, -193, -199, -210, -211, -224 UEGE-666, -696, -748, -751, -753, -826, -843, -845, -847, -852	α4
V134	I	27/33 (81.8%)	HG-02, -44, -51, -65, -66, -70, -150, -155, -162, -177, -179, -190, -193, -199, -210, -211, -224 UEGE-696, -748, -751, -752, -753, -843, -845, -846, -847, -852	α5
E140	G	6/33 (18.2%)	HG-51, -66, -162 UEGE-740, -753, -852	α5
T170	A	1/33 (3%)	UEGE-740	α5
A171	V	1/33 (3%)	HG-190	α5
T175	I	14/33 (42.4%)	HG-02, -44, -65, -150, -155, -162, -179, -190, -199 UEGE-748, -752, -826, -843, -846	α5
V181	I	1/33 (3%)	UEGE-640	α6
H200	Q	6/33 (18.2%)	HG-02, -44, -66, -177, -210 UEGE-753	α6
V203	I	1/33 (3%)	HG-43	α6
E210	K	2/33 (6%)	UEGE-740, -843	α6
R216	S	2/33 (6%)	HG-224, UEGE-740	α6
S221	N	2/33 (6%)	HG-02, -66	α6

### CagL polymorphisms and *vacA* genotypes of *H. pylori*

As mentioned before, polymorphisms in residues 58 and 59 have been associated with a higher risk of gastric disease, so we analyzed their relationship with the genotype of *vacA*. As seen in Table 3, 80.6% (25/31) of the strains carrying the *vacA* genotype s1/m1 had the polymorphism D58/K59, while of the five isolates with polymorphism N58, 100% had the genotype *vacA* s1/m1 regardless of the polymorphism in position 59. These results suggest that the polymorphism D58/K59 correlates with genotype *vacA* s1/m1.

**Table 3:** CagL polymorphisms and *vacA* genotypes of clinical isolates of *H. pylori*

CagL polymorphisms	*vacA* genotype			*p* value
	s1/m1 n = 31	s1/m2 n = 1	s2/m2 n = 1	
N58/E59	2 (6.5%)	0	0	
N58/K59	3 (9.7%)	0	0	
D58/K59	25 (80.6%)	1 (100%)	1 (100%)	< 0.05*
D58/E59	1 (3.2%)	0	0	

* Fisher’s exact test

### Phylogenetic relationship of CagL sequences

**Fig. 5:**
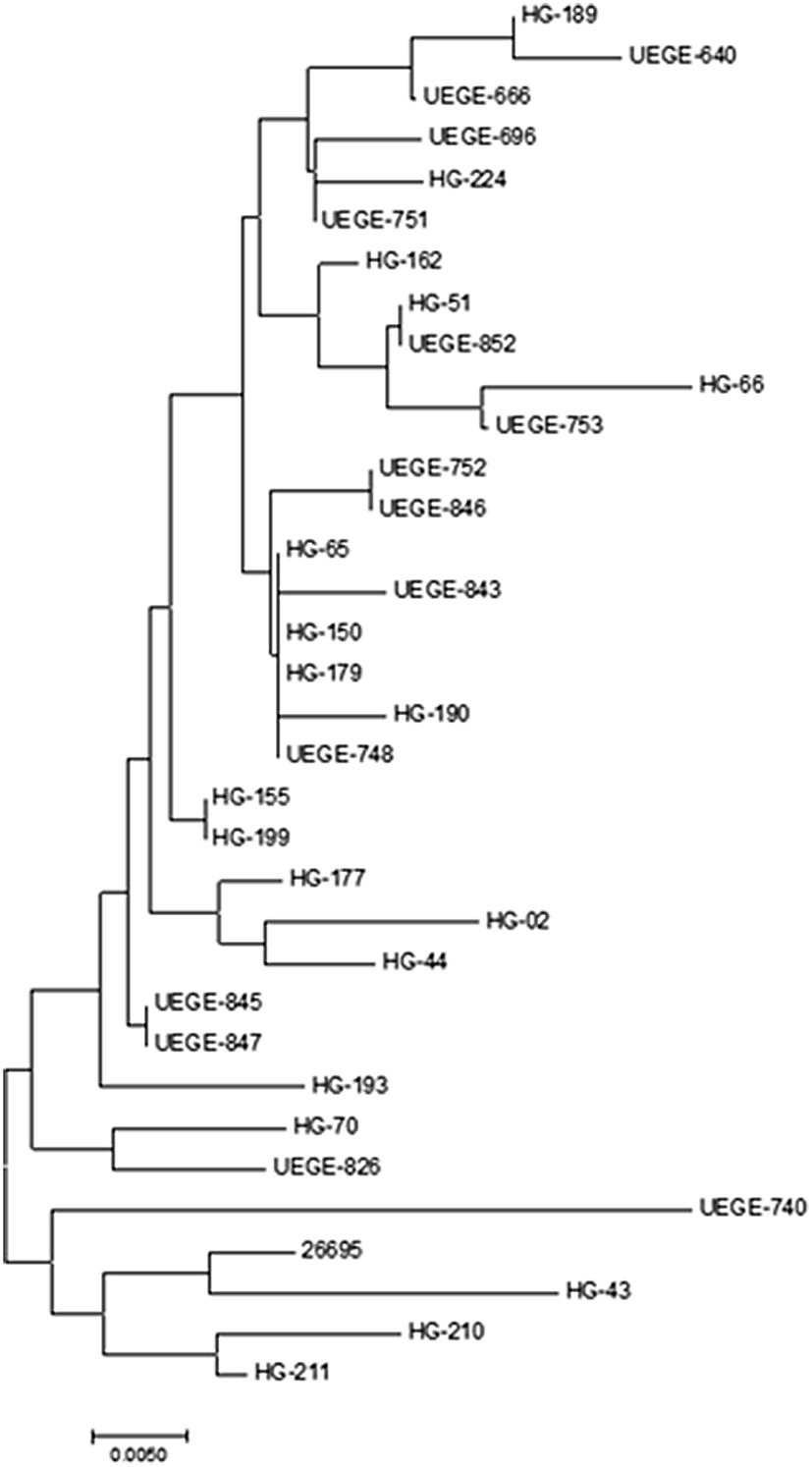
Phylogenetic tree of CagL sequences. Neighbor-Joining tree analysis of the CagL sequences of the 33 clinical isolates and strain ATCC 26695

Since we identified 4 polymorphic region sequences that have been found predominantly in Asian strains (NKMGQ, NEIGQ, DEIGQ, and DKIGK), we performed a phylogenetic analysis to determine if they are related. As shown in Fig. 5, at the bottom of the tree is a group formed by strains containing sequences NEIGQ and DEIGQ (HG-43, HG-210, and HG-211), and the reference strain ATCC 26695, which has the sequence NEMGE (found only in Europe). Related to this group is the strain carrying the sequence DKIGK (UEGE-740). Forming part of a second major group, which contains strains with sequences DKMGE and NKMGE, are the strains carrying the sequence NKMGQ. However, these are more related to the strain carrying the DKIGE sequence than to the rest.

## Discussion

Gastric diseases are of diverse etiology, and although chronic gastritis and more severe gastroduodenal disorders are attributed to *H. pylori*, Epstein–Barr virus and human Cytomegalovirus can also cause these diseases [25]. In this study we found that the prevalence of *H. pylori*, determined by bacterial isolation, was 30.5%. This percentage is similar to that reported by Román-Román et al. (47.8%) [26], but lower than that reported by other authors [Paniagua et al. (60.1%); Martínez-Carrillo et al. (77%)] [27, 28]. This variation in frequencies may be due to bias in the information provided by the patients; also, it is impossible to rule out that they have received antimicrobial treatment against *H. pylori* or any other infection in the recent past. Among the Mexican population self-medication is common and, although the sale of antibiotics without medical prescription is prohibited, in the state of Guerrero the metronidazole, an antiparasitic that is part of the treatment scheme for *H. pylori*, is available over the counter. Additionally, the probability of finding *H. pylori* in biopsies decreases as structural and functional changes appear in the infected mucosa. Another factor that may have contributed to the low frequency found in this work, is that the prevalence of *H. pylori* infection was determined by culture, which is a very specific method (95–100%) but not very sensitive (80–90%), since the results depend on the number of viable bacilli in the biopsy [29].

Among infected patients, the clinical outcome is determined by the genetic background and lifestyle of the host, as well as by *H. pylori* virulence factors. It has been proposed that the *H. pylori* virulence factor CagL has an important role in the pathogenesis of gastroduodenal diseases, and that its function can be determined by the amino acid sequence in specific positions [11, 12]. We analyzed the prevalence of *cagL* in *H. pylori* clinical isolates from Mexican patients with chronic gastritis, which was 72%. This is a lower percentage than those found in Asian patients, > 85% in Chinese, Indian, Iranian, Taiwanese and Malay patients [20, 21, 30, 31]; however, these patients had more severe diseases, like gastric cancer, peptic ulcer disease, and duodenal ulcer. Nevertheless, our result is higher to that found in Iranian patients with gastritis (64.2%) [32]. Apparently, the prevalence of *cagL* in clinical isolates from patients with gastritis is lower than that found in isolates from patients with peptic ulcer disease or gastric cancer. All our *cagA*^+^ isolates were *cagL*^+^, which is in accordance with previous reports that have shown that the prevalence of *cagL* is higher than, or very close to that of *cagA* [20, 30, 33].

We also analyzed the polymorphic region (residues 58 to 62) of CagL. We found that the most common polymorphism in position 58 corresponds to aspartic acid (D) (84.8%), followed by asparagine (N) (15.2%). It has been proposed that strains carrying aspartic acid at this position lead to a lower risk of gastric cancer in comparison with the asparagine carrying strains. In agreement with this, the polymorphism N58 was significantly associated with gastric cancer in Mexican patients from Mexico City [22, 32]. Regarding residue combinations in positions 58 and 59, polymorphism D58/K59 has been associated with gastric cancer in India, where patients carrying bacteria with this polymorphism are 3.8 times more likely to develop this disease [20]. However, our results showed that D58/K59 was the most common polymorphism in Mexican patients with chronic gastritis and it is associated with the *vacA* genotype s1/m1. We also found a high frequency of the polymorphisms M60 (85%), which has been previously shown to increase the chance of developing gastritis [32], and E62 (81.8%), which has been shown to be the most common polymorphism in strains from Iranian patients with chronic gastritis [32]. It is worth noting that in this region the only invariable residue is G61.

Besides the polymorphisms in the polymorphic region, Cherati et al. [32], reported other polymorphisms outside this region associated with different disease outcomes. They found that the polymorphism K122 had frequency of 88.8% in strains from patients with gastritis. Similarly, we found that the frequency of K122 in our isolates was 82%. In fact, 24/33 strains (73%) had the combination M60/K122. They also found that the frequency of polymorphism V134 is significantly higher in patients with gastric cancer than gastritis. In our strains, V134 had a prevalence of only 18.2%, being the most common the polymorphism I134 (81.8%). This result shows that in Mexican patients, polymorphism I134 is more common in chronic gastritis. We also identified two more residues with high variability, I73 and T175 (in *H. pylori* ATCC 26695). In strains isolated from Mexican patients with chronic gastritis, polymorphisms M73 and I175 have a frequency of 60.6% and 42.4%, respectively, however, in the literature there is no information regarding these residues or their association with clinical outcome, hence, the clinical significance of these results as well as the biological function of these polymorphisms need to be addressed in more detail. With respect to the I73 M mutation, isoleucine (I) and methionine (M) are nonpolar amino acids without charge, therefore, it is likely that this change has no important effects on the structure and function of CagL. The I175 mutation consists of the change of a polar threonine residue by one of nonpolar isoleucine, next to the TASLI motif and it is probable that this change influences the functions of CagL. It has been proposed that the TASLI region binds to integrin in an RGD-independent manner and it is probably that I175 residue contributes also to this function and to other functions of CagL. The TASLI motif is involved in binding to the cell, although only in the absence of another host cell ligands. Deletion of TASLI is related with a moderate reduction in IL-8 secretion and CagA translocation. In addition, it has been shown that the deletion of TASLI reduces de CagL binding to integrin [34].

Interestingly, the most common CagLHM sequence found in our study population was DKMGE, which is the most frequent worldwide, mainly in Africa and the American Continent. The second most frequent sequences were NEIGQ and NKMGQ. NEIGQ is also the second most common worldwide mainly in Europe, Asia, and North America, while NKMGQ has been found in Asia/Australasia but not in the American Continent. Additionally, we also found three sequences that are prevalent in East/Southeast Asia/Australasia (DKIGK), West/Central/South Asia (DEIGQ), and Central/South America (NKMGE) [23], as well as a new sequence not informed before (DKIGE). This sequence was found in only 1 clinical isolate (HG-193), and contains the sequence DKI, which is found mainly in strains from Asia/Australasia [23, 24, 35]. Despite of this, this isolate seems to be more related with those carrying sequence DKMGE, and in a lesser degree to strains carrying sequence NKMGQ (Fig. 5). These results show that the CagLHM sequences found in Mexican strains are diverse, with sequences not only common in our continent, but also in Asia, although all the CagA sequences are Western according to the EPIYA motifs [36].

The sequence diversity of CagL in the locally circulating strains may reflect the mobility and complex human interactions with the bacteria, but it may also be related to differences that modify the pathogenic function of the protein. More studies are needed on the diversity of CagL and the relationship of its polymorphisms with gastric diseases, as well as on the role of variations in the CagL hypervariable motif on the structure and function of the protein, and on the inflammatory process.

In the population of the state of Guerrero, Mexico, a high frequency of gastric diseases related to *H. pylori* persists, therefore, it is important to identify the virulence factors that confer a higher risk of developing serious diseases. The knowledge about these virulence factors will allow a better understanding of this process, facilitating the identification and characterization of biomarkers helpful in the detection of patients with greater risk of developing gastric cancer. It will also help to determine schemes and priorities of eradication treatments with anti–microbial compounds, as a measure to prevent gastric cancer.

## Conclusions

Most of the clinical isolates of *H. pylori* analyzed in this study were *cagL* positive, and all of them carried conserved RGD and RHS motifs. In Mexican patients with chronic gastritis, CagL polymorphisms D58, K59, M60, E62, K122, and I134 are the most common. CagLHM polymorphisms D58/K59 are related with the virulent genotype *vacA s1m1* of *H. pylori.*

## Methods

### Patients

We performed a cross-sectional study among 164 patients that attended to the Gastroenterology Service at the General Hospital Dr. Raymundo Abarca Alarcón, and to the Specialized Unit in Gastroenterology Endoscopy, both in Chilpancingo, Guerrero, Mexico. Patients who attended for an endoscopic study due to dyspepsia symptoms, and that have had no *H. pylori* eradication treatment during 1 month prior to the endoscopic procedure were selected. None of the patients included in this study were under treatment with proton pump inhibitors or with gastric pH neutralizing agents within 15 days prior to biopsy. Patients receiving non-steroidal anti-inflammatory therapy were excluded from the study. All patients signed a letter of consent. This project was approved by the Bioethics Committee of the Autonomous University of Guerrero, by the Department of Education and Research of the General Hospital Dr. Raymundo Abarca Alarcón, and by the authorized personnel of the Specialized Unit in Gastroenterology Endoscopy.

### Biopsies

The endoscopic study was performed after a fasting night with a video processor and video gastroscope (Fujinon, Wayne, NJ USA). Two biopsies were taken from the antrum of patients with chronic gastritis, one was immediately fixed in 10% formalin for histological examination, and the other one was placed in Brain Heart Infusion Broth (BHI) (Becton–Dickinson, North Carolina, USA) with 10% glycerol for the isolation of *H. pylori*. The biopsies were transported at 4 °C, and those intended for isolation of *H. pylori* were processed immediately.

### Isolation and identification of *H. pylori*

Each biopsy transported in BHI broth with 10% glycerol was macerated with a sterile wood applicator. Fifty microliters of the homogenates were cultivated on Columbia Agar plates (Becton–Dickinson, North Carolina, USA) supplemented with 10% ram blood, IsoVitaleX Enrichment and Helicobacter pylori selective supplement Dent (10 mg/L of vancomycin, 5 mg/L of trimethoprim, 5 mg/L of cefsulodin, 5 mg/L of amphotericin B) (Oxoid, Basingstoke, UK) at pH 6.8 to 7.0. The homogenates were distributed on the culture medium by isolation strip. The inoculated plates were incubated under microaerophilic conditions with 5% O_2_ and 5% CO_2_ at 37 °C in GasPak jars for 3–7 days. *H. pylori* was identified by colony morphology (small, transparent colonies, 1 mm in diameter), Gram staining and biochemical tests (urease, catalase and oxidase positive). *H. pylori* strain ATCC 43504 was used as positive control.

### Bacterial DNA extraction

Isolates identified as *H. pylori* were subcultured and incubated for 72 h. A pool of colonies from each isolate was resuspended in extraction solution (10 mM Tris pH 8, 10 mM EDTA, 0.5% SDS) for digestion with proteinase K. Total DNA was obtained by phenol: chloroform: isoamylic alcohol technique [37]. Total DNA concentration was determined using a NanoDrop 2000. DNA samples were stored at − 20 °C until use.

### Molecular confirmation and genotyping of *vacA* and *cagA* of *H. pylori* strains

Confirmation of *H. pylori* strains was done using oligonucleotides HP16SF and HPGR16SR (Table 4), which amplify a fragment of the 16S rRNA gene, according to the method described by Román-Román et al. [38]. *vacA* and *cagA* genotyping was assessed by multiple PCR with specific oligonucleotides VAIF and VAIR, VAGF and VAGR, and F1 and B1, respectively (Table 4). The reaction mixture contained 1.5 mM MgCl_2_; 0.2 mM dNTPs; 2.5 pmol of oligonucleotides F1 and B1, or 5 pmol of VAGF and VAGR, or 2.5 pmol of VAIF and VAIR; 1.5 U of Taq DNA polymerase (Invitrogen, Carlsbad, CA, USA) and 200 ng of DNA, in a total volume of 25 μL. Amplification conditions were: 1 cycle at 94 °C for 10 min; 35 cycles at 94 °C for 1 min, 57 °C for 1 min, 72 °C for 1 min; and a final extension cycle at 72 °C for 10 min. The PCR products were subjected to 2.5% agarose gel electrophoresis, stained with ethidium bromide and visualized with ultraviolet light (UV). In each PCR, DNA from strain ATCC 43504 (*vacA s1m1*/*cagA*^+^) was used as positive control, and as negative control DNA was replaced with sterile deionized water. All reactions were done in a Mastercycler Ep gradient thermal cycler (Eppendorf, Hamburg, Germany).

**Table 4:** Oligonucleotides used in this work

Gene	Oligonucleotide	Sequence 5′–3′	Amplicon size (bp)	References
*16S rRNA*	HP16SF	GCTAAGAGATCAGCCTATGTCC	522	[39]
	HPGR16SR	CAATCAGCGTCAGTAATGTTC		
*vacAs1*	VAIF	ATGGAAATACAACAAACACAC	259	[40]
*vacAs2*	VAIR	CTGCTTGAATGCGCCAAAC	286	
*vacAm1*	VAGF	CAATCTGTCCAATCAAGCGAG	570	[41]
*vacAm2*	VAGR	GCGTCTAAATAATTCCAAGG	645	
*cagA*	F1	GATAACAGGCAAGCTTTTGAGG	349	[42]
	B1	CTGCAAAAGATTGTTTGGCAGA		
Empty site	ESf	ACATTTTGGCTAAATAAACGCTG	360	[43]
	ESr	TCATGCGAGCGGCGATGTG		
*cagL*	cagL sense	GAAGATATAACAAGCGGTTT	651	[44]
	cagL antisense	TTTAACAATGATCTTACTTGA		
	cagL-Fwd-2	ACVAAGAGACCAACCARCAAG	165	This work
	cagL-16	TCGCTTCAAAATTGGCTTTC		[31]
	cagL-Fwd-2	ACVAAGAGACCAACCARCAAG	611	This work
	cagL-16	TTTAACAATGATCTTACTTGA		[44]

### Confirmation of *cag*PAI empty site

The absence of *cagA* and the pathogenicity island *cag*PAI in the *cagA*^−^ strains was confirmed by the empty-site assay by conventional PCR, using the ESf and ESr oligonucleotides (Table 4), which bind upstream and downstream, respectively, of the region where the *cag*PAI is inserted in the genome of the reference strain NCTC 12455 (NCTC: National Collection of Type Culture) [45]. The PCR mixture contained 50 ng of DNA, 0.08 mM dNTPs (Invitrogen, Carlsbad, CA, USA), 1 mM MgCl_2_, 5 pmol of each oligonucleotide and 1 U of Platinium Taq DNA Polymerase (Invitrogen, Carlsbad, USA), in a final volume of 15 μL. The amplification conditions were: 1 cycle at 94 °C for 5 min; 35 cycles at 94 °C for 30 s, 61 °C for 30 s and 72 °C for 45 s; and one final extension cycle at 72 °C for 7 min. PCR products were subjected to 2% agarose gel electrophoresis, followed by ethidium bromide staining and UV light observation. As negative control, DNA was replaced with sterile deionized water. DNA from strain ATCC 43504 (*cagA*^+^, *cag*PAI^+^) was used as a second negative control, and strain UEGE-644 (*cagA*^−^, *cag*PAI^−^) as positive control. The presence of a 360 bp product was considered indicative of the absence of *cagA* and *cag*PAI [43, 45].

### CagL amplification by PCR

A fragment of 651 bp from gene *cagL* (*hp0539*) was amplified by PCR using primers cagL sense and cagL antisense (Table 4). The reaction mixture had a final volume of 15 μL, and contained 2.5 mM MgCl_2_, 0.25 mM dNTP’s, 5 pmol of each oligonucleotide, 1 U of Taq recombinant DNA polymerase (Invitrogen, Massachusetts, USA) and 50 ng of DNA. The conditions used were: 1 cycle at 94 °C for 5 min; 45 cycles at 94 °C for 30 s, 55 °C for 30 s, and 72 °C for 1 min; and 1 cycle at 72 °C for 7 min. Each reaction included a positive (DNA from strain 26695) and a negative (DNA was substituted with water) control. All reactions were performed in a Mastercycler Ep gradient thermocycler (Eppendorf, Hamburg, Germany). PCR products were analyzed by agarose gel electrophoresis at 1.5% and stained with ethidium bromide. A second PCR was performed with DNA from those strains that were negative in the first reaction, using primers cagL-Fwd-2 and cagL-16, which amplified a 165 bp product (Table 4). The reaction mixture had a final volume of 15 μL, and contained 1.5 mM MgCl_2_, 0.25 mM dNTP’s, 5 pmol of each oligonucleotide, 1 U of Taq recombinant DNA polymerase (Invitrogen, Massachusetts, USA) and 50 ng of DNA. The conditions used were: 1 cycle at 94 °C for 5 min; 35 cycles at 94 °C for 45 s, 54 °C for 30 s, and 72 °C for 1 min; and 1 cycle at 72 °C for 7 min. Each reaction included a positive (DNA from strain 26695) and a negative (DNA was substituted with water) control. PCR products were analyzed by agarose gel electrophoresis at 1.8% and stained with ethidium bromide. A third PCR was performed with DNA from those strains that were negative in the first reaction, using primers cagL-Fwd-2 and cagL antisense (Table 4), in order to amplify a fragment of 611 bp suitable to upload in the GeneBank. The reaction mixture had a final volume of 25 μL and contained 1.5 mM MgCl_2_, 0.25 mM dNTP’s, 5 pmol of each oligonucleotide, 1 U of Taq recombinant DNA polymerase (Invitrogen, Massachusetts, USA) and 50 ng of DNA. The conditions used were: 1 cycle at 94 °C for 5 min; 35 cycles at 94 °C for 45 s, 56 °C for 30 s, and 72 °C for 45 s; and 1 cycle at 72 °C for 3 min. PCR products were analyzed by agarose gel electrophoresis at 1.8% and stained with ethidium bromide.

### Purification and sequencing of PCR products

PCR products were purified by the isopropanol method and sequenced in the Unidad de Síntesis y Secuenciación of the Instituto de Biotecnología, UNAM, using primers cagL sense and primer cagL-Fwd-2 (Table 4). All sequences were deposited in GenBank with accession numbers MG051618-MG051641 and MG214979-MG214987.

### Bioinformatic analysis

Nucleotide sequences were translated to aminoacidic sequences using the ExPASy Translate Tool (http://web.expasy.org/translate/). Multiple sequence alignment of translated sequences and phylogenetic analysis were performed using Molecular Evolutionary Genetics Analysis version 7.0 (MEGA7) software [46].

The evolutionary history was inferred using the Neighbor-Joining method [47]. The tree was drawn to scale, with branch lengths in the same units as those of the evolutionary distances used to infer the phylogenetic tree. The analysis involved 34 amino acid sequences of CagL.

## References

[1] Solnick JV, Tompkins LS (1992). *Helicobacter pylori* and gastroduodenal disease: pathogenesis and host–parasite interaction. Infect Agents Dis.

[2] Censini S, Lange C, Xiang Z, Crabtree JE, Ghiara P (1996). *cag*, a pathogenicity island of *Helicobacter pylori*, encodes type I-specific and disease-associated virulence factors. Proc Natl Acad Sci USA.

[3] Bridge DR, Merrell DS (2013). Polymorphism in the *Helicobacter pylori* CagA and VacA toxins and disease. Gut Microbes.

[4] Backert S, Tegtmeyer N, Fischer W (2015). Composition, structure and function of the *Helicobacter pylori cag* pathogenicity island encoded type IV secretion system. Future Microbiol.

[5] Kwok T, Zabler D, Urman S, Rohde M, Hartig R (2007). *Helicobacter* exploits integrin for type IV secretion and kinase activation. Nature.

[6] Backert S, Ziska E, Brinkmann V, Zimny-Arndt U, Fauconnier A (2000). Translocation of the *Helicobacter pylori* CagA protein in gastric epithelial cells by a type IV secretion apparatus. Cell Microbiol.

[7] Odenbreit S, Puls J, Sedlmaier B, Gerland E, Fischer W (2000). Translocation of *Helicobacter pylori* CagA into gastric epithelial cells by type IV secretion. Science.

[8] Pham KT, Weiss E, Jimenez Soto LF, Breithaupt U, Haas R (2012). CagI is an essential component of the *Helicobacter pylori* Cag type IV secretion system and forms a complex with CagL. PLoS ONE.

[9] Saha A, Backert S, Hammond CE, Gooz M, Smolka AJ (2010). *Helicobacter pylori* CagL activates ADAM17 to induce repression of the gastric H, K-ATPase alpha subunit. Gastroenterology.

[10] Wiedemann T, Hofbaur S, Loell E, Rieder G (2016). A C-terminal coiled-coil region of CagL is responsible for *Helicobacter pylori*-induced Il-8 expression. Eur J Microbiol Immunol.

[11] Barden S, Lange S, Tegtmeyer N, Conradi J, Sewald N (2013). A helical RGD motif promoting cell adhesion: crystal structures of the *Helicobacter pylori* type IV secretion system pilus protein CagL. Structure.

[12] Conradi J, Huber S, Gaus K, Mertink F, Royo Gracia S (2012). Cyclic RGD peptides interfere with binding of the *Helicobacter pylori* protein CagL to integrins alphaV beta3 and alpha5 beta1. Amino Acids.

[13] Wiedemann T, Hofbaur S, Tegtmeyer N, Huber S, Sewald N (2012). *Helicobacter pylori* CagL dependent induction of gastrin expression via a novel alphav beta5-integrin-integrin linked kinase signalling complex. Gut.

[14] Backert S, Fronzes R, Waksman G (2008). VirB2 and VirB5 proteins: specialized adhesins in bacterial type-IV secretion systems?. Trends Microbiol.

[15] Tegtmeyer N, Hartig R, Delahay RM, Rohde M, Brandt S (2010). A small fibronectin-mimicking protein from bacteria induces cell spreading and focal adhesion formation. J Biol Chem.

[16] Barden S, Niemann HH (2015). Adhesion of several cell lines to *Helicobacter pylori* CagL is mediated by integrin alphaV beta6 via an RGDLXXL motif. J Mol Biol.

[17] Conradi J, Tegtmeyer N, Wozna M, Wissbrock M, Michalek C (2012). An RGD helper sequence in CagL of *Helicobacter pylori* assists in interactions with integrins and injection of CagA. Front Cell Infect Microbiol.

[18] Tafreshi M, Zwickel N, Gorrell RJ, Kwok T (2015). Preservation of *Helicobacter pylori* CagA translocation and host cell proinflammatory responses in the face of CagL hypervariability at amino acid residues 58/59. PLoS ONE.

[19] Barden S, Schomburg B, Conradi J, Backert S, Sewald N (2014). Structure of a three-dimensional domain-swapped dimer of the *Helicobacter pylori* type IV secretion system pilus protein CagL. Acta Crystallogr D Biol Crystallogr.

[20] Shukla SK, Prasad KN, Tripathi A, Jaiswal V, Khatoon J (2013). *Helicobacter pylori cagL* amino acid polymorphisms and its association with gastroduodenal diseases. Gastric Cancer.

[21] Yeh YC, Chang WL, Yang HB, Cheng HC, Wu JJ (2011). *H*. *pylori cagL* amino acid sequence polymorphism Y58E59 induces a corpus shift of gastric integrin alpha5 beta1 related with gastric carcinogenesis. Mol Carcinog.

[22] Rizzato C, Torres J, Plummer M, Munoz N, Franceschi S (2012). Variations in *Helicobacter pylori* cytotoxin-associated genes and their influence in progression to gastric cancer: implications for prevention. PLoS ONE.

[23] Gorrell RJ, Zwickel N, Reynolds J, Bulach D, Kwok T (2016). *Helicobacter pylori* CagL hypervariable motif: a global analysis of geographical diversity and association with gastric cancer. J Infect Dis.

[24] Choi JM, Choi YH, Sudhanva MS, Devakumar S, Lee KH (2015). Crystal structure of CagL from *Helicobacter pylori* K74 strain. Biochem Biophys Res Commun.

[25] Del Moral-Hernandez O, Castanon-Sanchez CA, Reyes-Navarrete S, Martinez-Carrillo DN, Betancourt-Linares R (2019). Multiple infections by EBV, HCMV and *Helicobacter pylori* are highly frequent in patients with chronic gastritis and gastric cancer from Southwest Mexico: an observational study. Medicine.

[26] Roman-Roman A, Martinez-Carrillo DN, Atrisco-Morales J, Azucar-Heziquio JC, Cuevas-Caballero AS (2017). *Helicobacter pylori vacA* s1m1 genotype but not *cagA* or *babA2* increase the risk of ulcer and gastric cancer in patients from Southern Mexico. Gut Pathog.

[27] Paniagua GL, Monroy E, Rodriguez R, Arroniz S, Rodriguez C (2009). Frequency of *vacA*, *cagA* and *babA2* virulence markers in *Helicobacter pylori* strains isolated from Mexican patients with chronic gastritis. Ann Clin Microbiol Antimicrob.

[28] Martinez-Carrillo DN, Garza-Gonzalez E, Betancourt-Linares R, Monico-Manzano T, Antunez-Rivera C (2010). Association of IL1B -511C/-31T haplotype and *Helicobacter pylori vacA* genotypes with gastric ulcer and chronic gastritis. BMC Gastroenterol.

[29] Logan RP, Walker MM (2001). ABC of the upper gastrointestinal tract: epidemiology and diagnosis of *Helicobacter pylori* infection. BMJ.

[30] Schmidt HM, Andres S, Nilsson C, Kovach Z, Kaakoush NO (2010). The cag PAI is intact and functional but HP0521 varies significantly in *Helicobacter pylori* isolates from Malaysia and Singapore. Eur J Clin Microbiol Infect Dis.

[31] Raei N, Latifi-Navid S, Zahri S (2015). *Helicobacter pylori cag* pathogenicity island *cagL* and *orf17* genotypes predict risk of peptic ulcerations but not gastric cancer in Iran. Asian Pac J Cancer Prev.

[32] Cherati MR, Shokri-Shirvani J, Karkhah A, Rajabnia R, Nouri HR (2017). *Helicobacter pylori cagL* amino acid polymorphism D58E59 pave the way toward peptic ulcer disease while N58E59 is associated with gastric cancer in north of Iran. Microb Pathog.

[33] Yadegar A, Mobarez AM, Alebouyeh M, Mirzaei T, Kwok T (2014). Clinical relevance of *cagL* gene and virulence genotypes with disease outcomes in a *Helicobacter pylori* infected population from Iran. World J Microbiol Biotechnol.

[34] Bonig T, Olbermann P, Bats SH, Fischer W, Josenhans C (2016). Systematic site-directed mutagenesis of the *Helicobacter pylori* CagL protein of the Cag type IV secretion system identifies novel functional domains. Sci Rep.

[35] Ogawa H, Iwamoto A, Tanahashi T, Okada R, Yamamoto K (2017). Genetic variants of *Helicobacter pylori* type IV secretion system components CagL and CagI and their association with clinical outcomes. Gut Pathog.

[36] Atrisco-Morales J, Martinez-Santos VI, Roman-Roman A, Alarcon-Millan J, De Sampedro-Reyes J (2018). *vacA* s1m1 genotype and *cagA* EPIYA-ABC pattern are predominant among *Helicobacter pylori* strains isolated from Mexican patients with chronic gastritis. J Med Microbiol.

[37] Sambrook J, Maccallum P, Russel D (2001). Molecular cloning: a laboratory manual.

[38] Roman-Roman A, Giono-Cerezo S, Camorlinga-Ponce M, Martinez-Carrillo DN, Loaiza-Loeza S (2013). *vacA* genotypes of *Helicobacter pylori* in the oral cavity and stomach of patients with chronic gastritis and gastric ulcer. Enferm Infecc Microbiol Clin.

[39] Chang YH, Wang L, Lee MS, Cheng CW, Wu CY (2006). Genotypic characterization of *Helicobacter pylori cagA* and *vacA* from biopsy specimens of patients with gastroduodenal diseases. Mt Sinai J Med.

[40] Atherton JC, Cao P, Peek RM, Tummuru MK, Blaser MJ (1995). Mosaicism in vacuolating cytotoxin alleles of *Helicobacter pylori.* Association of specific *vacA* types with cytotoxin production and peptic ulceration. J Biol Chem.

[41] Yamaoka Y, Kodama T, Kita M, Imanishi J, Kashima K (1998). Relationship of *vacA* genotypes of *Helicobacter pylori* to *cagA* status, cytotoxin production, and clinical outcome. Helicobacter.

[42] Yamaoka Y, Kodama T, Gutierrez O, Kim JG, Kashima K (1999). Relationship between *Helicobacter pylori iceA*, *cagA*, and *vacA* status and clinical outcome: studies in four different countries. J Clin Microbiol.

[43] Akopyants NS, Clifton SW, Kersulyte D, Crabtree JE, Youree BE (1998). Analyses of the cag pathogenicity island of *Helicobacter pylori*. Mol Microbiol.

[44] Wang H, Huang S, Zhao J, Han J, Guan X (2013). Expression of CagL from *Helicobacter pylori* and preliminary study of its biological function. Indian J Microbiol.

[45] Slater E, Owen RJ, Williams M, Pounder RE (1999). Conservation of the *cag* pathogenicity island of *Helicobacter pylori*: associations with vacuolating cytotoxin allele and IS605 diversity. Gastroenterology.

[46] Kumar S, Stecher G, Tamura K (2016). MEGA7: molecular evolutionary genetics analysis version 7.0 for bigger datasets. Mol Biol Evol.

[47] Saitou N, Nei M (1987). The neighbor-joining method: a new method for reconstructing phylogenetic trees. Mol Biol Evol.

